# Vitamin C in Human Health and Disease

**DOI:** 10.3390/nu13051595

**Published:** 2021-05-11

**Authors:** Dariusz Nowak

**Affiliations:** Department of Clinical Physiology, Medical University of Lodz, Kosciuszki 4, 90-419 Lodz, Poland; dariusz.nowak@umed.lodz.pl

Although the symptoms related to vitamin C deficiency were known in ancient Egypt and eighteenth century Scottish surgeon James Lind found that scurvy (a disease resulting from insufficient dietary ingestion of vitamin C) could be effectively treated with citrus fruit, this vitamin was discovered only in the year 1912 and then after 21 years it was chemically synthetized and introduced to the market as the first vitamin supplement. Since that time, numerous activities of vitamin C have been discovered and described on the basis of experimental, interventional and epidemiological studies, and as their practical application in medicine was verified in randomized clinical trials. The Special Issue “Vitamin C in Human Health and Disease” includes 10 peer-reviewed papers (nine review articles and one original research article) which mainly focus on the broadly defined role of vitamin C in osteoporosis, sleep quality, exercise performance, oxidative stress and inflammatory response in hemodialyzed patients, the development of eye cataract and skin physiology. Two papers present the updated discussion on anti- and pro-oxidant effects of ascorbate and the effectiveness of high dose infusions in patients with advanced cancer. Patients with end stage renal insufficiency treated with renal replacement therapy are at high risk of vitamin C deficiency due to unintentional elimination of this vitamin during hemodialysis, and its increased utilization due to hemodialysis accompanied by inflammatory response and oxidative stress as well as decreased ingestion due to some dietary restrictions [1]. Administration of vitamin C, either oral or intravenous, normalized its circulating levels in hemodialyzed patients. This treatment was accompanied by reduction of the intensity of inflammatory processes and markers of systemic oxidative stress [1]. However, hemodialysis patients with a serum ferritin concentration higher than 500 µ/L may present with enhanced intensity of peroxidative processes after treatment with vitamin C. Similarly, intravenous coadministration of vitamin C with ferric iron may lead to aggravation of oxidative stress, resulting in an increase in circulating lipid and protein peroxidation markers [1]. On the other hand, some pro-oxidant activities of vitamin C, shown by observation of increased plasma levels and urinary excretion of 8-oxo-2-deoxyguanosine after treatment with this vitamin, can be beneficial in healthy subjects. This can secondarily increase the activity of DNA repair enzymes and removal of oxidatively modified deoxyribonucleosides [2]. The daily requirement of vitamin C depends on body mass, cigarette smoking, lifestyle, physical activity and health status. Moreover, the efficiency of the intestinal absorption of vitamin C is dependent on a variety of genetic factors (e.g., SLC23A1 and SLC23A2 genes) which, apart from dietary intake, may influence circulating vitamin C levels [3]. Ascorbic acid can have a positive effect on bone mineral density and some clinical observations suggest that a deficiency of vitamin C may be associated with the development of osteoporosis. Epidemiological studies showed a positive association between high dietary intake of vitamin C and bone mineral density [4]. Patients with bowel diseases (e.g., Crohn’s disease, ulcerative colitis) due to dietary restrictions (reduced consumption of fruits and vegetables) are at risk of vitamin C deficiency [3]. Although other dietary factors such as low intake of calcium and vitamin D can contribute to the development of osteoporosis, vitamin C seems to have an important protective effect, especially in bowel diseases accompanied by systemic oxidative stress [3,4]. However, clarification of the questions of whether administration of vitamin C can prevent the development of osteoporosis or could be helpful as adjuvant therapy needs the execution of numerous controlled clinical trials. Eyes and skin are exposed to UV light which may induce the generation of reactive oxygen species. They are attributed to the development of lens cataract, acceleration of skin aging and skin carcinogenesis. The concentration of vitamin C in the aqueous and vitreous humor of the eye is several-fold higher than that of plasma. Aqueous solutions of vitamin C can absorb UV light and inactivate reactive oxygen species, therefore ascorbate is recognized as the physiological sunscreen of the eye. Although a beneficial effect of vitamin C on skin is well documented, its role in the prevention of cataract is not. There is some evidence that ascorbate supplementation may prevent nuclear cataract occurring after vitrectomy but this still requires further studies [5]. High physical activity (e.g., strenuous and prolonged exercise) increases the daily requirement of vitamin C. Surprisingly, supplementation with vitamin C (even with high doses) did not result in improvement of physical performance in trained and untrained subjects of both sexes, while it had a positive effect on sleep quality (an opposite state to exercise) [6]. Several epidemiological studies and clinical trials revealed a positive association of daily intake of vitamin C with sleep duration, reduction of sleep disturbances and relief of symptoms of sleep movement disorders. Moreover, supplementation with ascorbate seems to reduce the dangerous consequences of sleep apnea such as oxidative stress and endothelial dysfunction [6]. There is a continuous debate on whether high-dose intravenously administered vitamin C has a beneficial effect in patients with advanced stage cancer. This question seems to be very important because the most frequent reason to introduce this treatment is the belief in its activity as a potent anti-cancer agent (based on results of experimental studies), its ability to augment the chemosensitivity of cancer cells and reduction of chemotherapy-related toxicities and fatigue. Moreover, a deficiency of vitamin C is frequent in this group of patients [7]. Unfortunately, the results of clinical trials did not prove that high-dose intravenously administered vitamin C has therapeutically significant anti-cancer activity and improves the effectiveness of conventional chemotherapy [7]. However, the possibility to reduce the toxicity of chemotherapy with infusions of vitamin C is a still open question due to the scarcity of suitable clinical studies. The only clinically positive effects of this therapy were the improvement of quality of life and relief of pain, fatigue, lack of appetite, nausea/vomiting and sleep disturbances. Therefore, intravenous administration of high doses of vitamin C could be rather considered as a part of palliative care but not as an anti-cancer therapy [7]. All these aforementioned topics as well as an association of vitamin C with periodontal disease and gout are extensively discussed in this Special Issue.

As a guest editor, I would like to thank all the authors for their effort to prepare interesting contributions and the reviewers for their constructive remarks and suggestions. I would also like to acknowledge persons of the publishing team of *Nutrients* who professionally cooperated with me in preparation of this Special Issue.

## References

[1] Chaghouri P., Maalouf N., Peters S.L., Nowak P.J., Peczek K., Zasowska-Nowak A., Nowicki M. (2021). Two faces of vitamin C in hemodialysis patients: Relation to oxidative stress and inflammation. Nutrients.

[2] Kaźmierczak-Barańska J., Boguszewska K., Adamus-Grabicka A., Karwowski B.T. (2020). Two faces of vitamin C-antioxidative and pro-oxidative agent. Nutrients.

[3] Ratajczak A.E., Szymczak-Tomczak A., Skrzypczak-Zielińska M., Rychter A.M., Zawada A., Dobrowolska A., Krela-Kaźmierczak I. (2020). Vitamin C deficiency and the risk of osteoporosis in patients with an inflammatory bowel disease. Nutrients.

[4] Brzezińska O., Łukasik Z., Makowska J., Walczak K. (2020). Role of vitamin C in osteoporosis development and treatment—A literature review. Nutrients.

[5] Lim J.C., Caballero-Arredondo M., Braakhuis A.J., Donaldson P.J. (2020). Vitamin C and the lens: New insights into delaying the onset of cataract. Nutrients.

[6] Otocka-Kmiecik A., Król A. (2020). The role of vitamin C in two distinct physiological states: Physical activity and sleep. Nutrients.

[7] Zasowska-Nowak A., Nowak P.J., Ciałkowska-Rysz A. (2021). High-dose vitamin C in advanced-stage cancer patients. Nutrients.

